# Adeno-associated virus capsid protein expression in *Escherichia coli* and chemically defined capsid assembly

**DOI:** 10.1038/s41598-019-54928-y

**Published:** 2019-12-09

**Authors:** Dinh To Le, Marco T. Radukic, Kristian M. Müller

**Affiliations:** Cellular and Molecular Biotechnology, Faculty of Technology, Bielefeld University, Bielefeld, Germany

**Keywords:** Genetic vectors, Viral proteins, Expression systems

## Abstract

Research and clinical applications of recombinant adeno-associated virus (rAAV) significantly increased in recent years alongside regulatory approvals of rAAV gene therapy products. To date, all rAAV vectors as well as AAV empty capsids are produced in eukaryotic cells. We explored a new route to generate AAV capsids with the aim to analyze capsid assembly in a chemically defined setting and pave the way for new production methods and applications based on AAV virus-like particles (VLPs). We generated these empty capsids by bacterial expression and subsequent concomitant protein refolding and VLP formation. AAV serotype 2 structural protein VP3 was expressed in *Escherichia coli*. VLPs formed as demonstrated by dynamic light scattering, atomic force microscopy, and ELISA. Furthermore, VLPs internalized into human HeLa cells. To extend the application range of the VLPs, we tested peptide insertions, at the genetic level, in a surface loop (amino acid position 587) or at the C-terminus of VP3 and these variants also formed VLPs. VLPs developed without assembly-activating protein (AAP), but adding purified recombinant AAP to the refolding process increased capsid yield. Our findings offer a new route to understand AAV assembly biology and open a toolbox for AAV production strategies that might enable capsid display for vaccination and matching of capsids with cargoes at large scale and low cost.

## Introduction

AAV is a member of the family of *Parvoviridae* genus *Dependoparvovirus* and consists of a single-stranded DNA (ssDNA) genome of 4.7 kb packed in a non-enveloped capsid of 60 proteins arranged in a T = 1 icosahedral symmetry. AAV serotype 2 (AAV2) is the best-studied member of the genus which comprises currently 13 human and primate serotypes^1^. The genome of AAV2 consists of two open-reading-frame (ORF) cassettes flanked by inverted terminal repeat (ITR) sequences. In the typical genome depiction, the left ORF cassette codes for four non-structural Rep proteins (Rep 78, Rep 68, Rep 52, and Rep 40), which are responsible for AAV DNA replication, transcriptional regulation, site-specific integration and packaging of DNA into the capsid^2–5^. The right ORF cassette codes for three capsid proteins VP1, VP2 and VP3 (VP proteins) with apparent molecular masses of 87 kDa, 73 kDa, and 62 kDa, respectively^6^. These proteins, which only differ in their N-terminus, are produced by alternative splicing and leaky scanning from one reading frame to achieve a molar ratio of VP1:VP2:VP3 = 1:1:10^7^. VP3 is the main structural protein and can form VP3-only capsids^8^. The right ORF cassette also codes in a different reading frame for the assembly-activating protein (AAP), which promotes capsid assembly by increasing capsid protein stability and VP-VP interactions^9,10^. The AAP of AAV2 (AAP2) also plays a role in transporting the capsid proteins to the nucleolus for assembly^11^. Notably, while the capsids of AAV4, AAV5 and AAV11 can assemble without AAP, the other AAV serotypes from 1–12 including AAV2 critically require AAP to form capsids^12^.

Virus-like particles (VLPs) assemble from structural proteins of viruses. They lack a genome and are thus non-replicating particles. In recent years, these particles attracted great interest for targeted therapeutic delivery and vaccination^13–15^. Previous studies showed that AAV empty capsids produced in HEK-293 cells can be modified to present epitopes for vaccination^16,17^. Moreover, the concept of AAV VLP production using a yeast expression system was introduced^18^.

AAV vectors have had increasing successes in recent clinical gene therapy trials. Among the AAV serotypes, AAV2 is a preferred model and its clinical suitability is highlighted by the approval as a vector in the drug Luxturna (Voretigene neparvovec) for the treatment of patients with an inherited form of vision loss by the Food and Drug Administration (FDA)^19^. Moreover, the drug Glybera (Alipogene tiparvovec) based on AAV1 was approved by the European Medicines Agency (EMA) in 2012^20^ and Zolgensma (Onasemnogene abeparvovec) based on AAV9 was approved by the FDA in 2019^21^. Mammalian cell (HEK-293) or insect cell (Sf9) based systems are the two most commonly used methods to produce rAAV. Despite their success, they also pose disadvantages. HEK-293 cell culture is difficult to scale, specifically when using adherent cells, post-translational modifications of rAAV lead to charge heterogeneity^22^, and foremost process- and product-related impurities occur^23^. Production in Sf9 cells has drawbacks related to the genetic instability of baculovirus stocks during the expansion phase, the difficulty to produce infectious AAV particles with a correct capsid protein ratio and the requirement to remove baculoviruses and its components^24,25^.

A rising number of therapeutic AAV applications requiring high AAV vector doses, such as tumor therapy^26^, present a challenge to current production methods. Clinical trials report dosing of 10^12^–10^13^ AAV genome copies per kg of body weight for liver transduction gene therapy^27^ and about 10^14^ genome copies/kg for targeting organs without porous capillary networks^28^ thus reaching acceptable cost limits of current production techniques. In this light, the less expensive host yeast has been explored for production. However, low vector yields hinder commercial deployment^29^. The production of AAV empty capsids in bacteria could be the first step of a new strategy for rAAV production, if later *in vitro* encapsidation of genomes becomes possible.

*E. coli*, the most important host for molecular biology, has been used successfully for the production of virus-like particles^13,14^. Notably, although distant from the original host, heterologous production of primate erythroparvovirus 1 (human parvovirus B19) and canine parvovirus VLPs, which also belong to family *Parvoviridae*, has been described in *E. coli*^30,31^.

With the long-term perspective to develop a novel method for rAAV production based on a prokaryotic host and the short-term goal to study AAV VLPs production in bacteria and analyze epitope presentation, we established AAV VP expression in *E. coli* and subsequent VLP formation. High-level expression of AAV2 VP3 capsid protein in *E. coli* and, for the first time, chemically-defined, concomitant refolding and assembly of VP3 protein into AAV capsids was achieved. Biological functionality of capsids was demonstrated by anti-capsid ELISA and imaging of cellular uptake.

## Results

### Expression and purification of VP3 proteins

**Figure 1:**
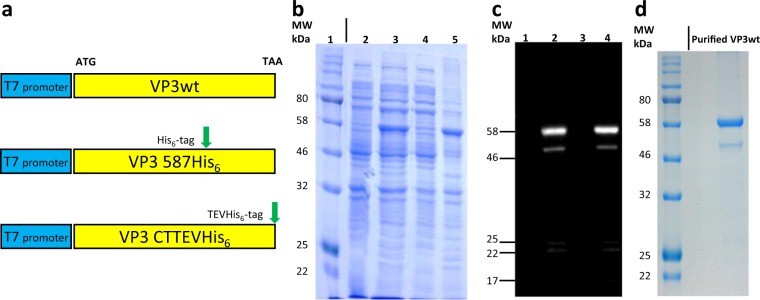
Expression of VP3wt protein in *E. coli*. (**a**) Schematic representation of VP3wt protein expression constructs downstream of a T7 promoter. Either a His-tag coding sequence was incorporated at the aa 587 coding position or a TEV_cleavage_site-His-tag coding sequence was cloned 3’ of the VP3wt gene. (**b**) Coomassie stained SDS-PAGE of VP3wt expression; lane 1, protein standard; lane 2, whole-cell protein of *E. coli* BL21(DE3) before induction; lane 3, whole-cell protein 18 h post-induction with IPTG; lane 4, soluble fraction of intracellular protein 18 h post-induction; lane 5, insoluble fraction of intracellular protein. (**c**) Western blot analysis; lane 1–4, samples corresponding to lane 2–5 (**b**) detected with B1 antibody. (**d**) SDS-PAGE of VP3wt protein after IEX purification. The black vertical line divides two different crops of the same gel. Full-length gels and blot are presented in Supplementary Fig. S1a–c.

AAV2 VP3 wild type (VP3wt) protein is known to form VP3-only capsids^8^. Hence, the codon usage of the VP3wt gene was optimized for *E. coli* and cloned as a synthetic gene into a pET vector downstream of a T7 promoter without an additional tag (Fig. 1a). We tested different expression conditions in shake flasks with the BL21(DE3) host, such as temperature (37 °C, 25 °C and 18 °C), OD at induction (OD_600_ of 0.6, 1.5 and 2.5) and duration of cultivation after induction (6 h, 18 h) to obtain soluble protein. However, only inclusion bodies were obtained due to suspected folding problems and growth-limiting effects of VP3wt protein to *E. coli*. Another expression system, which was subsequently evaluated, with a weaker lac promoter and *E. coli* RV308 as host also yielded inclusion bodies (Supplementary Fig. S1d). Consequently, we settled with overexpression with the T7 system in inclusion body form at 18 °C and induction of expression at an OD_600_ of 1.5 for 18 h (Fig. 1b, lane 5). The identity of the VP3 protein was confirmed by Western blot analysis using the antibody B1. Interestingly, in addition to a strong band at the expected size of VP3wt (theoretical mass 60.06 kDa), the B1 antibody identified four weaker and smaller bands. This suggests that VP3wt is unstable during expression (Fig. 1c, lane 2 and lane 4). Capsid proteins were then solubilized under denaturing condition (8 M Urea) and purified by denaturing anion-exchange chromatography (IEX). SDS-PAGE analysis of purified protein showed a major protein band at about 60 kDa which is in agreement with the theoretical mass of VP3wt (Fig. 1d). The purified protein yield with one degradation band around 50 kDa was around 10 mg per liter of culture with an estimated final purity of the correct-mass protein of 79% (by SDS-PAGE).

### Assembly and characterization of VLPs

We used VP3wt proteins in denaturing buffer as the starting material for refolding and concomitant *in vitro* assembly to VLPs. In other studies, capsid formation from denatured protein was initiated by the removal of denaturant^30,32^. To test this strategy, the VP3wt sample, which was concentrated and re-buffered in denaturing buffer with 5 M guanidine/HCl and 1 mM DTT as reducing agent, was dialyzed against PBS buffer containing 0.2 mol/l L- arginine at different pH conditions (pH 6, pH 7.5, pH 8.5, and pH 9). Significant amounts of VP3wt protein precipitated at pH 6 and pH 7.5. At pH 8.5 and pH 9, however, precipitation decreased. At these pH values, after removal of aggregates by centrifugation and filtration, the yield of soluble protein was around 36% at pH 8.5 and 70% at pH 9. Since particles aggregated when going back to the neutral pH (Supplementary Fig. S1e), the samples were used at pH 9 for further characterization.

**Figure 2:**
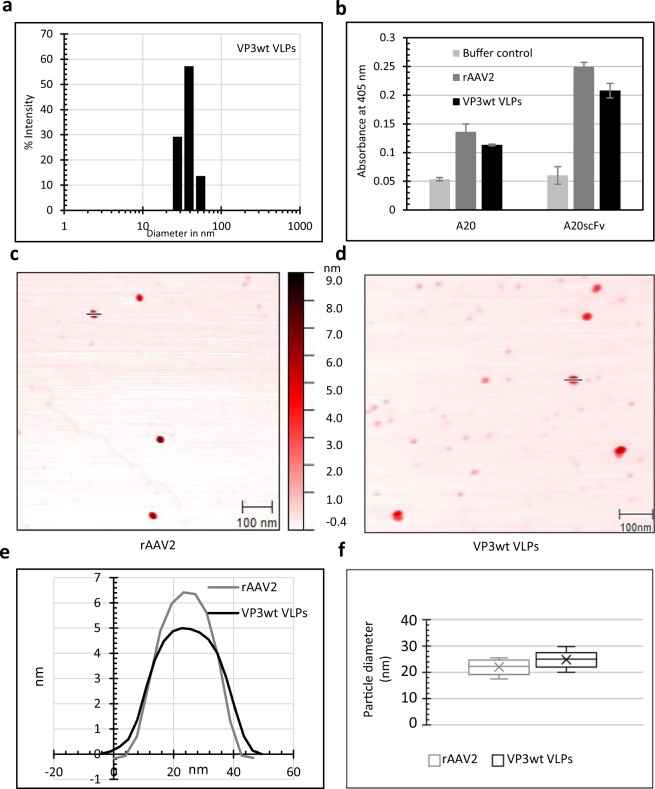
Characterization of VP3wt VLPs. (**a**) Size distribution of VP3wt VLP determined by DLS at pH 9.0. (**b**) ELISA probed with anti-intact-capsid AAV2 antibodies (A20 mAb or A20 scFv-Fc); samples were rAAV2 particles (1.6 × 10^9^ particles/ml) and VP3wt VLPs (50 µg/ml). (**c,d**) AFM images of rAAV2 and VP3wt VLPs, respectively. Scale bars are 100 nm. (**e**) AFM height profiles of rAAV2 and VP3wt VLPs. (**f**) Box plot of the size distribution of rAAV2 particles and VP3wt VLPs determined by AFM (n = 9, mean ± SD, box from first to third quartile, cross median marker). Full AFM images are presented in Supplementary Fig. S2a,b.

First, the presence of VLPs and/or their intermediate forms was analyzed by dynamic light scattering (DLS) at pH 9. The dialyzed VP3wt sample showed a mean hydrodynamic diameter of 37.7 nm and a polydispersity of 0.22 indicating homogeneity and the assembly of VLPs (Fig. 2a). Note that the hydrodynamic size is always greater than the physical size, and that this result is consistent with the hydrodynamic size of rAAV measured by DLS from other reports (34.4 nm or 38.2 nm)^33,34^.

Next, the VP3wt VLP conformation was assessed by ELISA with the A20 monoclonal antibody (mAb), which only binds to assembled AAV2 capsids^35^. Parallel to the A20 antibody, we also used a recombinant A20 single-chain Fv fragment Fc fusion antibody variant (A20 scFv-Fc) cloned in our lab and produced in HEK-293 cells (K. Teschner *et al*., in preparation). The ELISA signals indicated correctly assembled VLPs (Fig. 2b). We compared the ELISA result to a sample of rAAV2 with known capsid concentration and estimated the ratio of correctly assembled particles to be 1 VLP per 5000 theoretical VLPs based on VP3wt concentration (i.e. protein concentration divided by 60). The total yield from 0.23 mg denatured protein was 5 × 10^9^ correctly assembled VLPs in 1.5 ml.

The physical size and height profile of the particles were measured by atomic force microscopy (AFM). Recombinant AAV2 produced in HEK-293 cells was used for comparison. The shape and diameter of rAAV2 and VP3wt VLPs were similar, spherical and around 22 nm in diameter (Fig. 2c–f), which is expected for AAV^36^. In the AFM images of the *in-vitro* assembly also particles of a smaller diameter and higher-order aggregates were observed (Supplementary Fig. S2b).

### Cellular uptake of VP3wt VLPs

**Figure 3:**
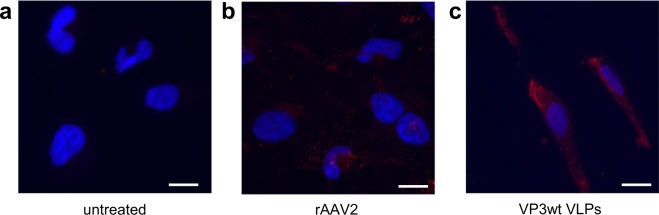
Fluorescent microscopy images of internalization. HeLa cells were incubated with either (**a**) medium, (**b**) rAAV2 (3 × 10^4^ particles/cell) or (**c**) VP3wt VLPs (final concentration of 50 µg/ml) at 37 °C. After 2 h, cells were fixed, permeabilized and stained with the A20 antibody followed by a DyLight 594 conjugated secondary antibody (red) as well as with DAPI (blue) and imaged with a 40 × objective. Scale bars are 10 µm. Full images are presented in Supplementary Fig. S3.

We used HeLa cells to evaluate cellular uptake and thereby biological activity of VP3wt VLPs, as these cells are known to have a high density of AAV2’s primary receptor heparan sulfate proteoglycan^37^. In this experiment, HeLa cells were incubated with rAAV2 or VP3wt VLPs, fixed, permeabilized and the A20 antibody as well as a fluorescent scondary antibody (red fluorescent signal) were added for detection with fluorescence microscopy (Fig. 3). No red fluorescent background signal was seen in the buffer control treated with the detection antibody (Fig. 3a). The distribution of rAAV2 was shifted towards the nucleus (Fig. 3b), while VP3wt VLPs were widely distributed in the cytoplasm, potentially in endosomes (Fig. 3c). We attribute this to the known nuclear translocation role of VP1, however, most of rAAV2 particles were still located in the cytoplasm after 2 h. This result indicates that VP3wt VLPs are biologically active and can internalize into cells.

### Effect of AAP2 on *in vitro* assembly

**Figure 4:**
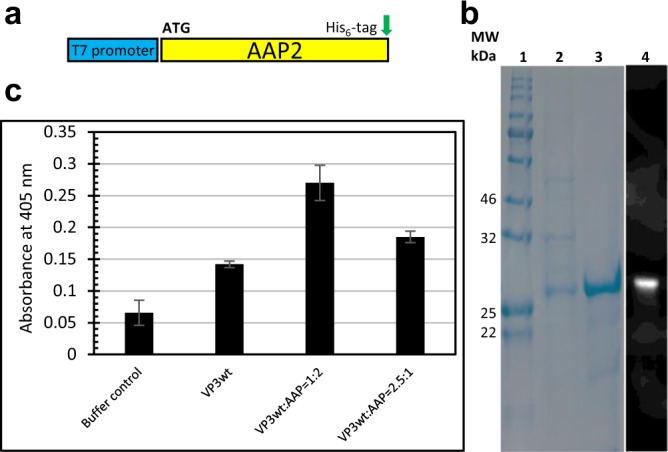
Impact of AAP2 on *in vitro* assembly. (**a**) Schematic representation of AAP2 expression construct. A His-tag coding sequence was added 3′of the AAP2 gene. (**b**) Coomassie SDS-PAGE; (1) protein standard; (2) insoluble fraction of intracellular protein before purification; (3) IMAC purified protein; (4) Western blot of the insoluble fraction detected with anti-His-tag antibody. (**c)** ELISA after *in vitro* assembly using the same final protein concentration (50 µg/ml) with different ratios of VP3wt:AAP2. Full-length gel and blot are presented in Supplementary Fig. S4.

AAP2 plays a critical role in AAV2 assembly. To investigate the impact of AAP2 on the production of AAV2 VLPs *in vitro*, we expressed AAP2 in *E. coli*. For the purpose of detection and purification, we added a C-terminal His-tag (Fig. 4a), as a modification at this position does not interfere with AAP function^38^. As for VP3, we tested various expression temperatures, such as 37 °C, 25 °C and 18 °C, to acquire AAP2 in soluble form. At 18 °C, AAP2 was expressed as a soluble protein, however, most proteins degraded during expression (Supplementary Fig. S5). Therefore, we settled with an inclusion body production at 37 °C and convenient purification by immobilized metal ion affinity chromatography (IMAC) under denaturing condition (Fig. 4b). Under these conditions, AAP was stable. We observed an apparent molecular weight of AAP2 of about 28 kDa, which is consistent with AAP2 produced in HEK-293 cells^12^. AAP2 was then added to the *in-vitro* assembly reaction in the presence of 5 M guanidine with VP3wt:AAP2 ratios of 1:2 or 2.5:1 and the refolding and assembly was performed as described. After dialysis, analysis by ELISA revealed that even though VP3wt can form capsids on its own, AAP2 enhanced the *in-vitro* assembly (Fig. 4c). Specifically, at a ratio of VP3wt:AAP2 of 1:2, the ELISA signal of VLP particles was 1.9 times greater than that of the VP3wt-only assembly reaction. This suggests that the yield of *in vitro* assembly increases in the presence of AAP2.

### Surface modification but not C-terminal extension is compatible with capsid antibody A20 mAb detection

**Figure 5:**
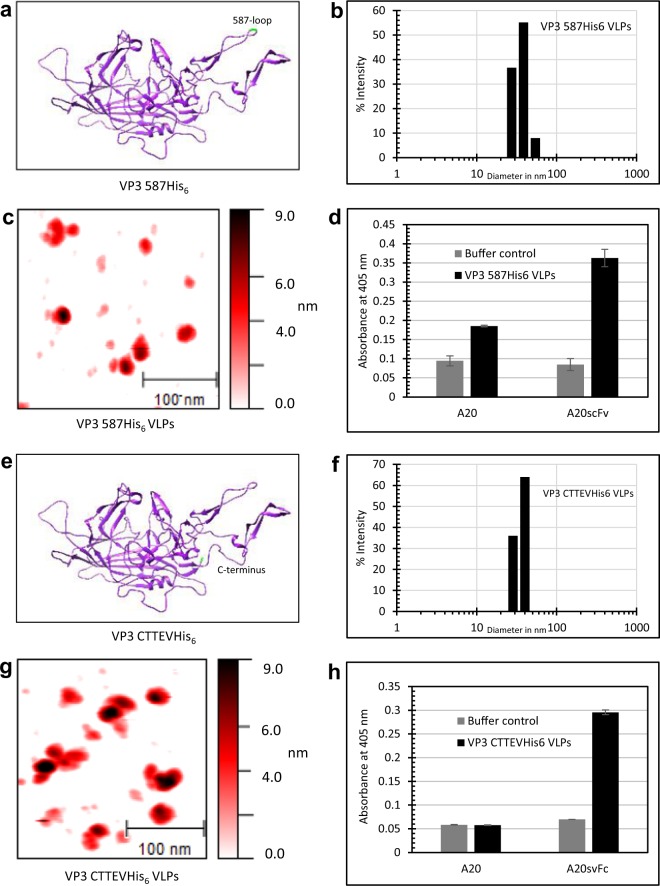
The effect of incorporation of a small peptide, either into the 587-loop or at the C-terminus of VP3wt, on *in vitro* assembly. (**a,e**) Representations of VP3wt protein structure (PDB ID: 1LP3) with highlighted 587-loop and C-terminus. (**b,f**) DLS size distribution of VP3 587His_6_ VLPs (35.7 ± 7.5 nm) and VP3 CTTEVHis_6_ VLPs (35.9 ± 5.5 nm). (**c,g**) AFM images of VP3 587His_6_ VLPs and VP3 CTTEVHis_6_ VLPs. Scale bars are 100 nm. (**d,h**) ELISA of modified VLPs using either A20 mAb or A20 scFv-Fc antibody for detection. Full AFM images are presented in Supplementary Fig. S2c,d.

Incorporation of peptides into the capsid is an important strategy in AAV retargeting^39^ and also gains interest in vaccine development^16,17^. To evaluate the effect of small peptide insertions on *in vitro* assembly, we chose two sites for modification. We genetically incorporated either a tag containing a TEV protease cleavage site followed by a His-tag at the C-terminus (VP3 CTTEVHis_6_) or a His-tag into the 587-loop (VP3 587His_6_) of VP3wt protein (Fig. 5a,e). The two sites were previously described to tolerate insertions *in vivo*^39,40^. We found that neither insertion of the tag at the C-terminus nor the 587-loop affected protein expression in *E. coli*. *In vitro* assembly of both modified proteins formed VLPs according to DLS and AFM measurements (Fig. 5b,c,f,g).

In ELISA, the anti-capsid A20 antibody did recognize VP3 587His_6_ particles as did the A20 scFv-Fc recombinant antibody construct (Fig. 5d) indicating wt-identical VLP assembly. Interestingly, ELISA results also showed that the A20 monoclonal antibody did not bind to VP3 CTTEVHis_6_ VLPs although particles were present (Fig. 5h). In contrast, the A20 scFv-Fc readily recognized VP3 CTTEVHis_6_ VLPs. These findings suggest a more flexible binding mode of this antibody construct. The results show that the 587-loop of VP3 is a potential position for capsid modification of particles assembled in a chemically-defined reaction.

## Discussion

AAV emerged as the most prominent candidate for gene therapy approaches. However, the several steps of AAV capsid assembly still remain unclear. AAV assembly has been proposed to occur in two steps. Empty capsids form and are then filled with its single-stranded DNA genome^5^. Hence, production of empty capsids from *E. coli* might not only help to elucidate the AAV biology aspect in capsid assembly, but also provide a feasible strategy for future AAV production. Production of rAAV2 capsids *in vitro* was first reported by Steinbach *et al*.^41^. The group used the recombinant baculovirus strategy to separately express and purify capsid proteins. AAV2 capsids then formed only in the presence of HeLa cell extract. In our study, capsid proteins were produced in *E. coli* and capsids formed in a chemically defined assembly reaction. To our knowledge, this is the first experimental evidence for true *in vitro*, cell-free assembly of AAV capsids.

*E. coli* remains one of the most attractive hosts to produce recombinant proteins because of well-established protocols and low cost. Due to the high level of expression in *E. coli*, heterologous proteins tend to aggregate and form inclusion bodies with a lack of biological activity. For proteins that can be refolded *in vitro*, however, especially for toxic or unstable proteins or proteins with potential protease activity, inclusion body formation can be advantageous due to the high volumetric yield and easy purification^42^. In addition, viral particles forming in *E. coli* might encapsidate bacterial polymers, such as DNA or RNA, which are then difficult to remove and not desired for human application. In our study, VP3 protein expression blocked *E. coli* growth at physiological temperatures and no useful amounts of VP3 were obtained in soluble form even at low temperatures. However, VP3 was effectively expressed in inclusion bodies and subsequently easily purified by a one-step purification, either IEX or IMAC. This was our preferred method. We confirmed the presence of VP3 during expression with the B1 antibody, which recognizes a C-terminal epitope of VP3^35^. Next to the major VP3 band, additional lower molecular weight bands were detected in Western blots. We suppose that VP3 is proteolytically degraded, possibly by an intrinsic protease activity as found in AAV2 particles^43^, or cleaved by *E. coli* proteases.

*In vitro* assembly of icosahedral VLPs is a complex process, which depends on various factors such as temperature, pH, ionic strength and the presence of additives^30,31,41^. In the current study, we used PBS containing 0.2 M L- arginine with different pH values to optimize the assembly reaction. L- arginine aids to form VLPs *in vitro* due to its ability to increase protein solubility and prevent aggregation of intermediate forms^44^. In previous studies, the pH showed significant impact on *in vitro* assembly of VLPs^31,32^. In the present study, the majority of VP3wt protein precipitated at pH 6 and pH 7.5. This can be explained with the theoretical isoelectric point of VP3 of pH 6.40 (ProtParam Expasy Webtool) to 6.88 (Geneious R9 Software). To enhance the solubility of particles, we chose basic conditions with pH 8.5 and pH 9 for further study. We found a suitable assembly condition to be PBS containing 0.2 M L- arginine at pH 9. This indicates that charge plays a significant role during *in vitro* assembly. Furthermore, VP3wt contains five free cysteines, some located in proximity and at the inter-protein interface^45^, which likely need to get buried during folding and assembly to avoid oxidation and/or crosslinking. This is particular critical for the thiolate anions present at levels above pH 8. We used DTT as reducing agent in the denaturing buffer but not the dialysis buffer. Keeping a reducing agent for the first dialysis steps might further reduce aggregation.

We characterized our assembly results by DLS and observed an average diameter in line with the expected hydrodynamic diameter. AFM imaging (Figs. 2c,d; 5c,g) showed particles with proper shape and diameter. In addition, we found other particles with a smaller diameter, which we attribute to assembly intermediates. The mechanism of capsid assembly of icosahedral virus is known to involve the formation of intermediate forms, and our observation correlates with those obtained for *in vitro* production of other icosahedral capsids^30,46^. Also, larger structures are visible, which might have formed during the drying procedure on the mica support.

VP3wt assembled capsids were also confirmed by ELISA, for which we used the well-established A20 antibody that specifically recognizes a conformational epitope on the AAV2 capsid exterior^35^. Additionally, we routinely used an A20 single-chain Fv-Fc variant for capsid detection, which gave comparable results. However, the structural requirement seemed to be less strict.

AAV2 binds to permissive cells using heparan sulfate proteoglycan (HSPG)^47^ and then a universal AAV receptor, which was identified with high-affinity to multiple AAV serotypes including AAV2^48^. These receptor-virus interfaces all lie within the VP3-only capsid. After attachment to the cell membrane, AAV2 internalizes by receptor-mediated endocytosis. Endosomal escape and nuclear entry, the next steps in infection, are attributed to the VP1 capsid protein^49,50^. Therefore, VP3wt VLPs should be able to enter cells that express the corresponding receptors but should not be able to translocate to the nucleus. We show that 2 h after transduction with the given buffer conditions, VP3wt VLPs internalized and distributed granular in the cytosol without nuclear preference, which is compatible with expectations of endosomal confinement. rAAV2 from mammalian production showed a similar appearance albeit with a shift towards the nucleus. These observations are consistent with a previously described distribution of rAAV2 and rAAV2 with deleted VP1 after infection of HeLa cells^51^, which supports the finding of biological activity of our VLPs. Further work will elucidate the internalization pathway of the particles.

Moreover, our study provides evidence that AAV2 VP3 proteins can form capsids *in vitro*, even in the complete absence of AAP2. AAP2 likely acts as a chaperone and/or a scaffold that directly or indirectly binds the lumenal surface of VP proteins and plays a role in VP stability and in VP oligomerization during assembly^10,52^. However, the exact mode of action of AAP2 in AAV2 assembly remains unclear. Our initial results of AAP2-free assembly agree with the previous report from Steinbach *at al*.^41^, although the authors of this study used HeLa cell extract for assembly. Here, by using a chemically defined reaction, we present direct evidence that the ability to form a capsid is entirely a property of VP3. To further investigate the role of AAP2 in capsid assembly, we expressed it in *E. coli* as inclusion bodies. Subsequent addition of solubilized AAP to our assembly reaction resulted in a 1.9-fold increase of mAb A20 positive particles over the AAP2-free assembly reaction. Although the complexity of the experiment provides a challenge for interpretation, the result indicates that AAP2 directly aides in capsid assembly, next to its before described function in VP stabilization by inhibition of proteasomal, lysosomal or autophagosomal degradation and nuclear translocalization^10,11^.

Finally, we explored possibilities to assemble capsids from modified VP3 proteins. Our loop and C-terminal insertions did not interfere with *in vitro* assembly as detected by DLS and AFM and gave VLP yields comparable to that of wild type VP3. As the capsid is known to tolerate these modifications *in vivo*^39,40,53^, our result hints that the *in-vitro* assembly process in general reflects the cellular process. Since the loop-region is exposed on the capsid surface, this opens the possibility to produce VLPs with modified tropism. Furthermore, these modified VLPs could be used to display functional proteins and act as antigen carriers for vaccination as described before for other virus particles^14,15^. However, we note that VLPs with VP3 C-terminal modification were detectable only by the A20 scFv-Fc, but not by the monoclonal IgG3-class A20 antibody. The broader binding of the single-chain construct can possibly be attributed to its general structural flexibility, allowing binding of slightly distorted capsids. One group found that a VP3 C-terminal His-tag is compatible with biological function^40^ and another found that the tag interferes with capsid assembly *in vivo*^54^. From our data, we can conclude that a C-terminal His-tag is compatible with forming particles, which are very similar in shape and diameter to wt capsids, but show subtle structural differences revealed by A20 mAb recognition.

In summary, the current study shows that AAV2 VP3 proteins can be expressed in *E. coli*, and that these proteins are able to form AAV2 capsids in a defined *in-vitro* reaction. This work provides a tool to assess capsid assembly of AAV under controlled conditions. At the same time, it opens the door for *in vitro* AAV capsid production with wide applications in vaccine development, cellular delivery of small-molecule drugs and possibly genetic payloads in gene therapy. Further studies will improve the assembly yield and elucidate the mechanisms of *in vitro* assembly.

## Methods

### Expression and purification of proteins

VP3wt was expressed in *E. coli* BL21(DE3) from a chemically synthesized, codon-optimized sequence (GeneArt/ThermoFisher Scientific, gene and amino acid sequence given in Supplementary Information subsection 2.1 and 2.2) inserted into the *Nde*I and *Xho*I sites of pET24b (Novagen/Merck). Transformed *E. coli* was cultured in LB medium containing Kanamycin (50 µg/ml) at 37 °C to an OD_600_ of 1.5, then isopropyl β-D-thiogalactopyranoside (IPTG) was added to a concentration of 0.4 mM, and the culture was incubated at 18 °C for additional 18 h. Cells were harvested at 5,000 × g, 4 °C for 15 min. Pelleted cells were re-suspended in a lysis buffer (50 mM NaH_2_PO_4_, 300 mM NaCl, pH 8) supplemented with 1 mg/ml lysozyme and lysed by sonication on ice (Branson Sonifier 250 with micro tip, power setting 70%, constant duty, 10 cycles, 30 s each). The recombinant proteins formed inclusion bodies, therefore, cell debris and inclusion bodies were collected by centrifugation at 10,000 × g, 4 °C for 20 min. The inclusion bodies were washed three times with lysis buffer. The remaining pellet was solubilized in ion-exchange chromatography (IEX) buffer A (20 mM Tris-HCl, 8 M Urea, pH 8). A 2 ml Q Sepharose resin (GE Healthcare) packed column was equilibrated with IEX Buffer A. The column was then loaded with sample and washed with IEX Buffer A. Bound proteins were eluted with a gradient of IEX Buffer B (20 mM Tris-HCl, 1 M NaCl, 8 M Urea, pH 8). Fractions containing target proteins were collected and concentrated with centrifugal filter units with a 30 kDa molecular weight cut-off. In this step, the buffer was changed to another denaturing buffer (5 M GuHCl, 20 mM Tris-HCl, 0.15 M NaCl, 1 mM EDTA, 1 mM DTT, pH 8).

Other VP3 constructs were designed to additionally code for either a TEV protease cleavage site-His_6_-tag fused to the C-terminus or a His_6_-tag at the amino acid position 587 (gene and amino acid sequences given in Supplementary Information subsection 2.3 to 2.6). The protein expression methods were used as described above. Protein AAP2 was also constructed into vector pET24b with a His_6_-tag at the C-terminus. This protein was expressed with 0.4 mM IPTG induction at 37 °C for 4 h and purified by immobilized metal-ion affinity chromatography (IMAC). Briefly, a column with 2 ml of Ni-NTA Agarose (Macherey-Nagel) was equilibrated with IMAC buffer A (50 mM NaH_2_PO_4_, 300 mM NaCl, 10 mM imidazole, 8 M Urea, pH 8), then sample was loaded and washed with five column volumes of IMAC buffer A containing 7% IMAC buffer B (50 mM NaH_2_PO_4_, 300 mM NaCl, 250 mM imidazole, 8 M Urea, pH 8). Afterwards, bound proteins were eluted with 100% IMAC buffer B.

### SDS-PAGE and western blot

Samples of protein were separated on a 12% polyacrylamide (12% T, 0.4% C) with a 4% collection gel, followed by semi-dry transfer to nitrocellulose membranes (88018, ThermoFisher Scientific, 0.45 µm). After blocking with 10% (w/v) non-fat dry milk (Applichem) in TBS buffer (500 mM Tris-HCl, 1.5 M NaCl, pH 7.5), the membranes were incubated 1 h at room temperature with either anti-AAV VP1/VP2/VP3 mouse monoclonal, B1 antibody (1: 100 dilution) (Progen) for detecting VP3 proteins or anti-His-tag antibody (Tetra·His, Qiagen) for detecting AAP2 protein. Detection was achieved using anti-mouse antibody coupled to horseradish peroxidase (Goat anti-Mouse IgG (H + L) Secondary Antibody, HRP, 1: 2500 dilution) (31430, ThermoFisher Scientific), SuperSignal™ West Pico PLUS Chemiluminescent Substrate (34580, ThermoFisher Scientific) and a Fusion FX camera system (Vilber).

### *In vitro* assembly of VLPs

Capsid assembly was carried out by dialyzing 1.5 mL of VP3 proteins (0.15 mg/mL) in solubilization buffer five times against 100 mL of phosphate buffered saline (PBS) (500 mM NaCl, 100 mM KCl, 10 mM Na_2_HPO_4_, 10 mM KH_2_PO_4_, pH 9) containing 0.2 M L- arginine, at 4 °C, changing buffer every 12 h over a 60-h period. To investigate the effect of AAP2 on *in vitro* assembly, purified VP3 protein (0.15 mg/ml) was mixed with a varying concentration of purified AAP2 (a VP3:AAP2 ratio of 1:2 or 2.5:1). Afterwards, capsid assembly was performed as mentioned above.

### DLS, AFM Characterization of VLPs

Dynamic light scattering (DLS) was performed on a DynaPro99 (Wyatt Technology) instrument. Samples were filtered through 0.22 µm PVDF syringe filters (Millipore) and centrifuged at 18,000 × g for 30 min before measurement. Measurements for each sample were averages of at least 20 acquisitions.

Atomic Force Microscopy (AFM) measurements of rAAV2 and VLPs were carried out using a Multimode 8 AFM (Bruker) with Tap300Al-G cantilevers (BudgetSensors) in tapping mode in air. Samples after filtration and centrifugation were spotted onto freshly cleaved mica and incubated for one minute. The mica was then briefly rinsed with water and dried under a gentle nitrogen flow. The analysis of the AFM images was performed with Gwyddion 2.49 software. The diameter of visualized particles was measured at half maximum particle height. The rAAV used as control was provided by K. Teschner and was produced using a three plasmid system in HEK-293 cells and purified by affinity chromatography.

### ELISA

VLPs were detected by ELISA with anti-AAV2 (intact particle) mouse monoclonal, A20 antibody or its single-chain derivative A20 scFv-Fc. MaxiSorp 96 well-plates (Nunc) were coated with VLPs (50 µg/ml) or rAAV2 (1.6 × 10^9^ AAV2/ml) for 1 h at 37 °C and afterwards blocked with 0.8% BSA for 1 h. Samples were incubated with A20 antibody (Progen, 1: 250 dilution), or A20 scFv-Fc (produced by our working group, 1: 250 dilution, manuscript in preparation) for another 1 h at 37 °C. Anti-mouse-HRP (31430, ThermoFisher Scientific) or anti-human-HRP (ThermoFisher Scientific) (1: 2000, 1 h incubation at 37 °C) was used for detection. Following every incubation step, the plate was washed three times with 0.05% Tween in blocking buffer. Lastly, samples were developed with 1 g/L 2,2′-Azinobis [3-ethylbenzothiazoline-6-sulfonic acid]-diammonium salt (ABTS), and optical density was measured by a microplate reader (BioTek) at 405 nm.

### Cellular uptake of VP3wt VLPs

To study cellular uptake of VLPs, HeLa cells were seeded on poly-L-lysine-coated 12-mm glass coverslips placed in a 24-well plate at a density of 3 × 10^4^ cells/well in DMEM 10% FCS and allowed to adhere overnight. Then, VP3wt VLPs (50 µg/ml, estimated 1.4 × 10^4^ particles/cell) and rAAV2 (3 × 10^4^ particles/cell) were added and incubated for 2 h. Following three washes with PBS, cells were fixed with 4% paraformaldehyde for 15 min at room temperature. The cells were then permeabilized with 0.2% Triton X-100 in PBS buffer for 5 min. Afterwards, the permeabilized cells were blocked in 1% BSA in PBS buffer for 30 min at room temperature. The cells were incubated with primary antibody (A20 antibody, Progen (1:40)) overnight at 4 °C, followed by incubation with the second antibody (Goat anti-Mouse IgG (H + L) Secondary Antibody, DyLight 594, 35510, ThermoFisher Scientific (1:500)) for 1 h at room temperature. After three washes in PBS, the cells were incubated in 10 µM 4′,6-diamidino-2-phenylindole (DAPI) for 10 min at room temperature. Coverslips were then mounted on glass slides with mounting medium (Mowiol-Dabco) and cells were imaged on a Leica DMI6000 B microscope.

## Supplementary information

SI: Adeno-associated virus capsid protein expression in Escherichia coli and chemically defined capsid assembly
