# Supplementary material for: Adeno-associated virus capsid protein expression in Escherichia coli and chemically defined capsid assembly
Source: Sci Rep. 2019 Dec 9;9:18631. doi: 10.1038/s41598-019-54928-y (PMC6901487; doi:10.1038/s41598-019-54928-y)
Supplement: Supplementary file 1 — SI: Adeno-associated virus capsid protein expression in Escherichia coli and chemically defined capsid assembly [file 41598_2019_54928_MOESM1_ESM.pdf]

Supplementary information

Adeno-associated virus capsid protein expression in *Escherichia coli* and chemically defined capsid assembly

Dinh To Le[1], Marco T. Radukic[1], Kristian M. Müller[1]*

[1] Cellular and Molecular Biotechnology, Faculty of Technology, Bielefeld University, Bielefeld, Germany

* Corresponding author: Kristian M. Müller, Cellular and Molecular Biotechnology, Bielefeld University, Universitätsstraße 25, 33615 Bielefeld, Germany; Email: kristian@syntbio.net

SI contents

1 Supplementary figures 2
2 VP3 sequences 5
2.1 VP3wt coding sequence 5
2.2 VP3wt amino acid sequence 6
2.3 VP3 587His$_6$ coding sequence 6
2.4 VP3 587His$_6$ amino acid sequence 6
2.5 VP3 CTTEVHis$_6$ coding sequence 7
2.6 VP3 CTTEVHis$_6$ amino acid sequence 7

1 Supplementary figures

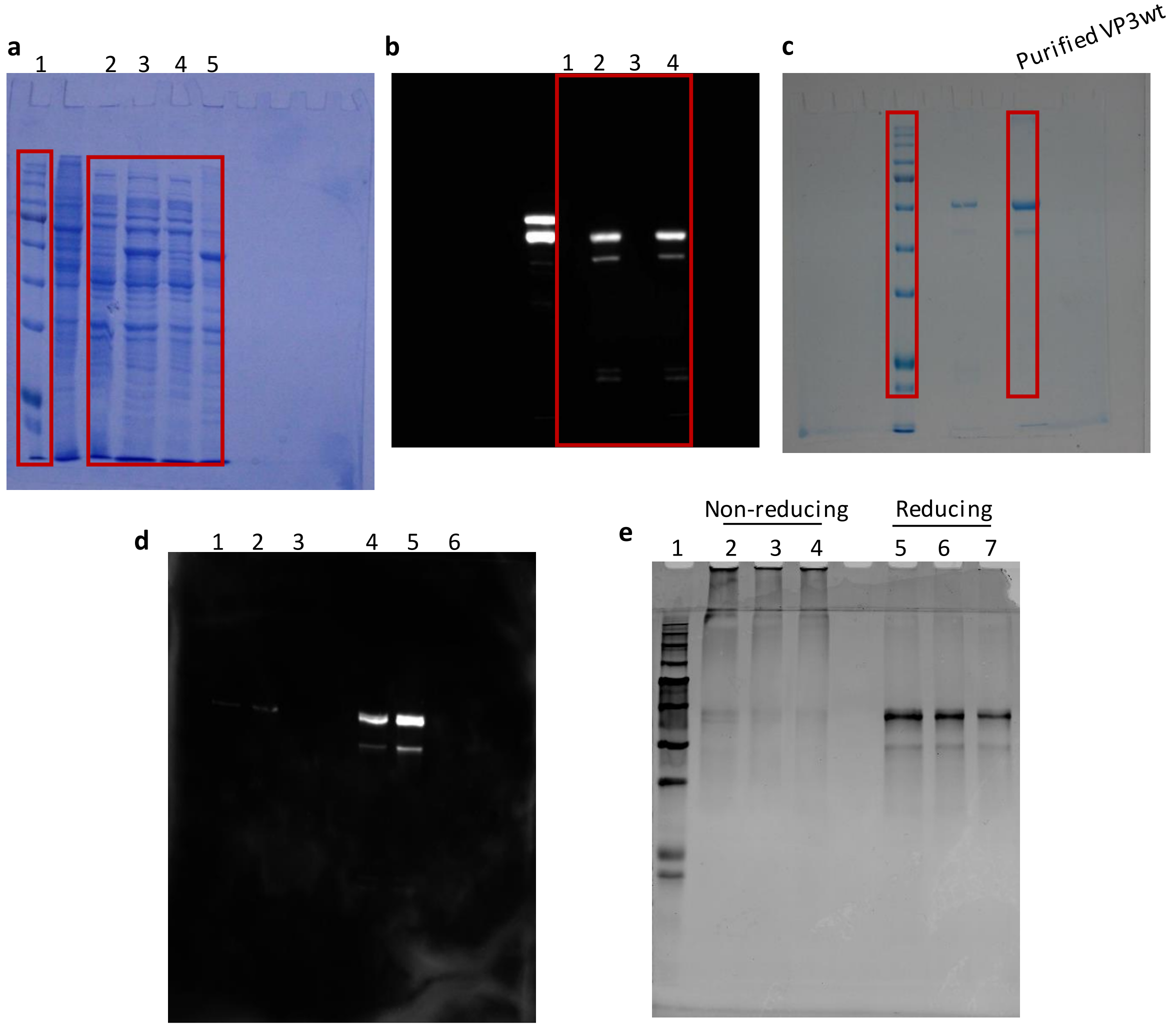

Supplementary Figure S1: Full-length gels and blots. (a), (b), (c) correspond to Fig. 1b, c, d in the manuscript. The red rectangles indicate cropped parts of the gels and blot. (d) Western blot analysis of VP3wt expression using plasmid pAK100 in *E. coli* RV308 at 18°C and 37°C; lane 1, whole-cell protein of *E. coli* RV308 post-induction with IPTG at 18°C; lane 2, insoluble fraction of intracellular protein post-induction with IPTG at 18°C; lane 3, soluble fraction of intracellular protein post-induction with IPTG at 18°C; lane 4, whole-cell protein of *E. coli* RV308 post-induction with IPTG at 37°C; lane 5, insoluble fraction of intracellular protein post-induction with IPTG at 37°C; lane 6, soluble fraction of intracellular protein post-induction with IPTG at 37°C. (e) Non-reducing and reducing SDS-PAGE of particles at pH 9 and samples after changing from pH 9 to pH 7.4 (before and after removal of aggregates); lane 1, protein standard; lane 2, particles at pH 9 in non-reducing condition; lane 3, 4, samples after change from pH 9 to pH 7.4, before and after removal of aggregates by centrifugation in non-reducing condition, respectively; lane 5, particles at pH 9 in reducing condition, lane 6 and 7, samples after changing from pH 9 to pH 7.4, before and after removal of aggregates by centrifugation in reducing conditions, respectively. We assume that the significant disulfide crosslinking seen in the gel occurs during sample boiling in SDS under non-reducing conditions.

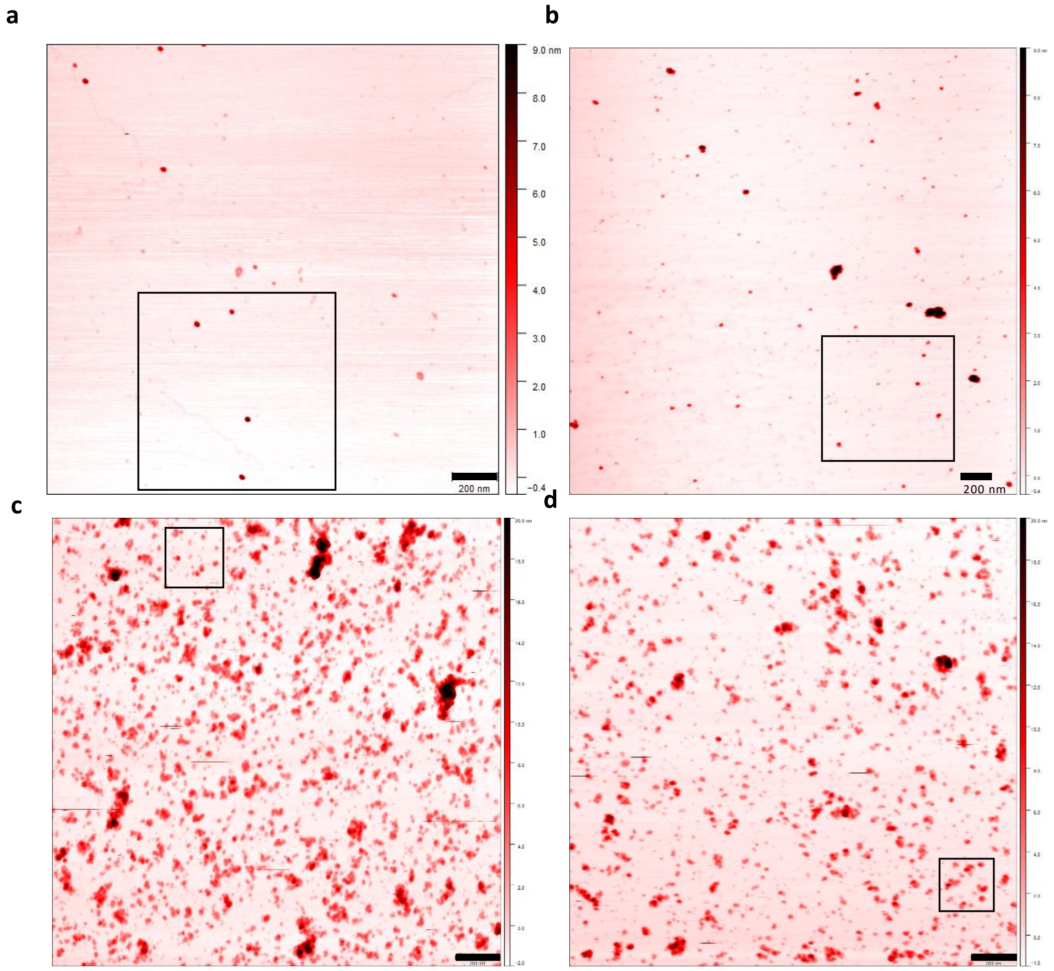

Supplementary Figure S2: Atomic force micrographs of (a) rAAV2, (b) VP3wt VLPs, (c) VP3 587His$_6$ VLPs (c) and (d) VP3 CTTEVHis$_6$ VLPs. The scale bars indicate 200 nm. (a), (b) correspond to Fig. 2c, d in the manuscript; (c), (d) correspond to Fig. 5c, g in the manuscript. The black squares indicate cropped parts of the images.

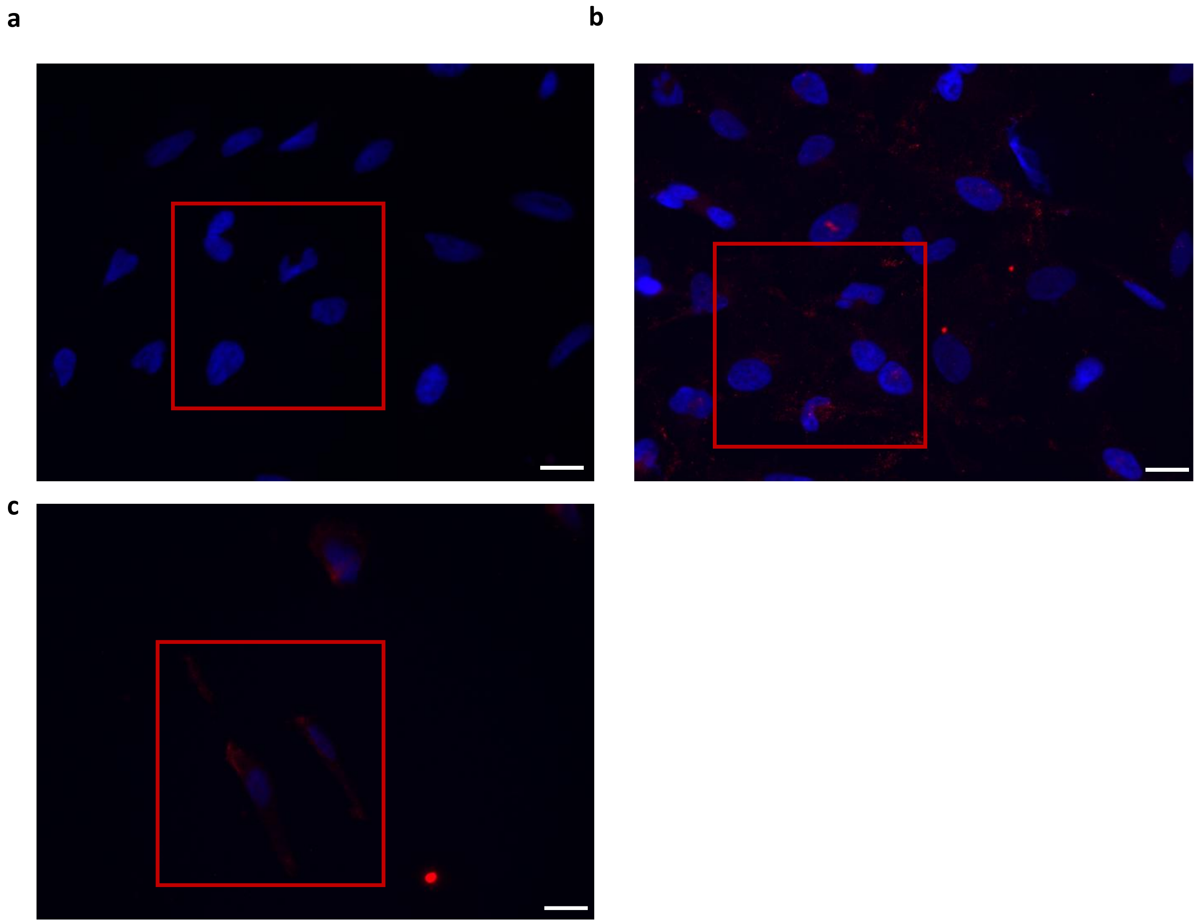

Supplementary Figure S3: Fluorescent microscopy of HeLa cells treated with rAAV2 or VP3wt VLPs. (a) untreated; (b) rAAV2; (c) VP3wt VLPs. All images show the overlay of rAAV2 or VP3wt VLPs (red) and nuclei (blue) channels. The scale bars indicate 25 µm. (a), (b), (c) correspond to Fig. 3a, b, c in the manuscript. The red squares indicate cropped parts of the images.

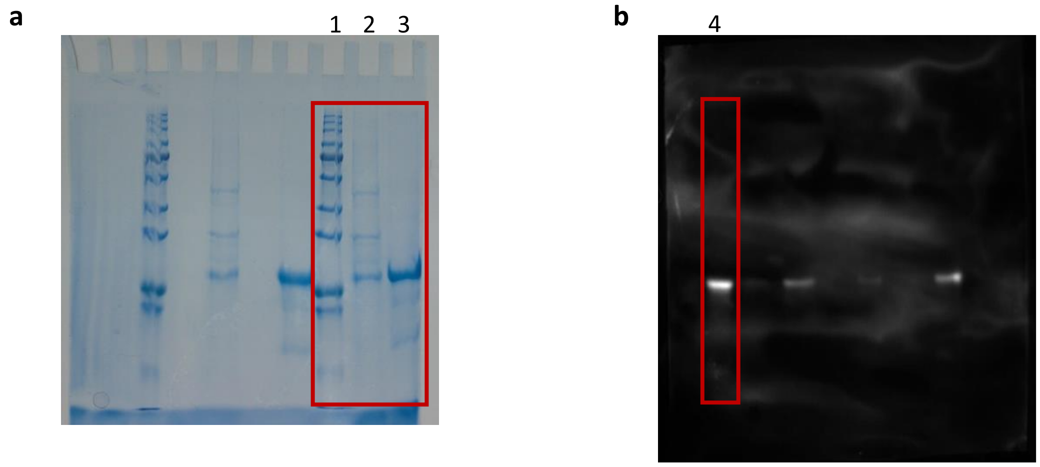

Supplementary Figure S4: Full-length gel and blot of AAP2 expression in *E. coli* at 37°C. (a), (b) correspond to Fig. 4b in the manuscript. The red rectangles indicate cropped parts of the gel and blot.

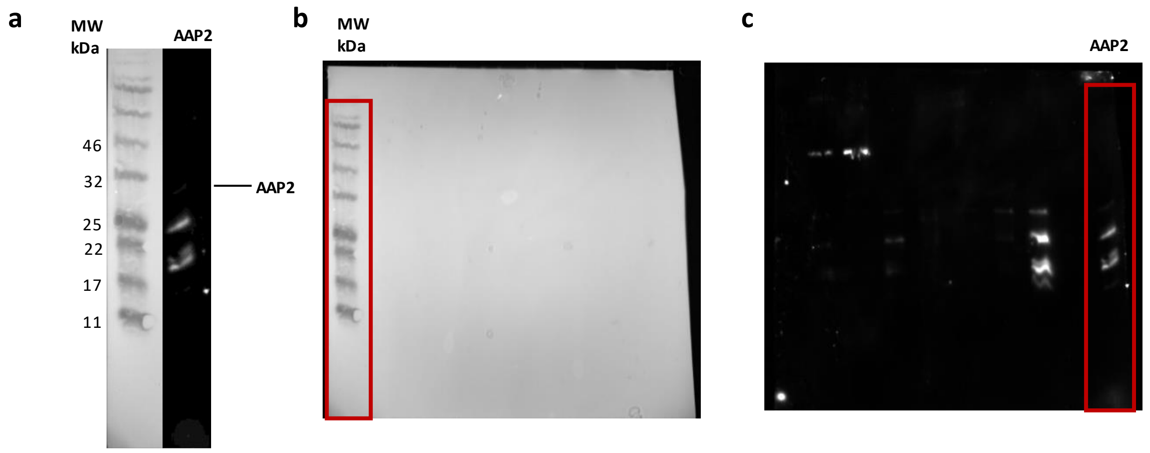

Supplementary Figure S5: (a) Expression of AAP2 in *E. coli* at 18°C. The AAP2-degraded bands were detected by anti-His tag antibody. (b), (c) Full-length blots of AAP2 expression in *E. coli* at 18°C. The red rectanglels indicate cropped parts of the blots.

2 VP3 sequences

2.1 VP3wt coding sequence

ATGGCAACCGGTTCAGGCGCGCCAATGGCAGATAATAATGAAGGTGCAGATGGTGTTGGTAATAGCAGCGGT
AATTGGCATTGTGATAGCACCTGGATGGGTGATCGTGTTATTACCACAAGCACCCGTACCTGGGCACTGCCGA
CCTATAATAACCATCTGTATAAACAAATTAGCAGCCAGAGCGGTGCAAGCAATGATAATCATTATTTTGGTTAT
AGCACCCCGTGGGGCTATTTTGATTTTAATCGTTTTCACTGCCATTTCAGTCCGCGTGATTGGCAGCGTCTGATT
AATAACAATTGGGGTTTTCGTCCGAAACGCCTGAACTTTAAACTGTTTAATATCCAGGTGAAAGAAGTGACCCA
GAACGACGGCACCACCACCATTGCAAATAATCTGACCAGCACCGTTCAGGTTTTTACCGATAGCGAATATCAGC
TGCCTTATGTTCTGGGTAGCGCACATCAGGGTTGTCTGCCACCGTTTCCGGCAGATGTTTTTATGGTTCCGCAG
TATGGTTATCTGACCCTGAATAATGGTAGCCAGGCAGTTGGTCGTAGCAGCTTTTATTGTCTGGAATATTTTCC
GAGCCAGATGCTGCGTACCGGTAATAACTTTACCTTTAGCTATACCTTTGAGGATGTGCCGTTTCATAGCAGCT
ATGCACATAGCCAGAGCCTGGATCGTCTGATGAATCCGCTGATTGATCAGTATCTGTATTATCTGAGCCGTACC
AATACACCGAGCGGTACAACCACACAGAGCCGTCTGCAATTTAGTCAGGCAGGCGCAAGCGATATTCGTGATC
AGAGCCGTAATTGGCTGCCTGGTCCGTGTTATCGTCAGCAGCGTGTTAGCAAAACCAGCGCAGATAACAATAA
CAGCGAATATAGTTGGACCGGTGCCACCAAATATCATCTGAATGGTCGTGATAGCCTGGTTAATCCGGGTCCT
GCAATGGCCAGCCATAAAGATGATGAAGAAAAATTCTTTCCGCAGAGTGGCGTTCTGATTTTTGGTAAACAGG
GTAGCGAAAAAACCAACGTGGATATCGAAAAAGTGATGATCACCGATGAAGAAGAGATTCGTACCACCAATC
CGGTTGCGACCGAACAGTATGGTAGCGTTAGCACCAATCTGCAACGTGGTAATCGTCAGGCAGCAACCGCAG
ATGTTAATACCCAGGGTGTTCTGCCTGGTATGGTTTGGCAGGATCGTGATGTTTATCTGCAAGGTCCGATTTGG
GCAAAAATTCCGCATACCGATGGTCATTTTCATCCGAGTCCGCTGATGGGTGGTTTTGGTCTGAAACATCCGCC
TCCGCAGATTCTGATTAAGAATACTCCGGTTCCGGCAAATCCGAGCACCACCTTTAGCGCAGCAAAATTTGCCA
GCTTTATTACCCAGTATAGTACCGGTCAGGTTAGCGTTGAAATTGAATGGGAACTGCAAAAAGAAAACAGCAA
ACGTTGGAATCCGGAAATTCAGTATACCAGCAACTATAACAAAAGCGTGAACGTGGATTTTACCGTGGATACC
AATGGTGTTTATAGCGAACCGCGTCCGATTGGCACCCGCTATCTGACACGTAATCTGTAA

2.2 VP3wt amino acid sequence

MATGSGAPMADNNEGADGVGNSSGNWHCDSTWMGDRVITTSTRTWALPTYNNHLYKQISSQSGASNDNHYF
GYSTPWGYFDFNRFHCHFSPRDWQRLINNNWGFRPKRLNFKLFNIQVKEVTQNDGTTTIANNLTSTVQVFTDSEY
QLPYVLGSAHQGCLPPFPADVFMVPQYGYLTLNNGSQAVGRSSFYCLEYFPSQMLRTGNNFTFSYTFEDVPFHSSY
AHSQSLDRLMNPLIDQYLYYLSRTNTPSGTTTQSRLQFSQAGASDIRDQSRNWLPGPCYRQQRVSKTSADNNNSEY
SWTGATKYHLNGRDSLVNPGPAMASHKDDEEKFFPQSGVLIFGKQGSEKTNVDIEKVMITDEEEIRTTNPVATEQY
GSVSTNLQRGNRQAATADVNTQGVLPGMVWQDRDVYLQGPIWAKIPHTDGHFHPSPLMGGFGLKHPPPQILIK
NTPVPANPSTTFSAAKFASFITQYSTGQVSVEIEWELQKENSKRWNPEIQYTSNYNKSVNVDFTVDTNGVYSEPRPI
GTRYLTRNL

2.3 VP3 587His_6 coding sequence

the 587-loop insertion of His_6 is in boldface

ATGGCAACCGGTTCAGGCGCGCCAATGGCAGATAATAATGAAGGTGCAGATGGTGTTGGTAATAGCAGCGGT
AATTGGCATTGTGATAGCACCTGGATGGGTGATCGTGTTATTACCACAAGCACCCGTACCTGGGCACTGCCGA
CCTATAATAACCATCTGTATAAACAAATTAGCAGCCAGAGCGGTGCAAGCAATGATAATCATTATTTTGGTTAT
AGCACCCCGTGGGGCTATTTTGATTTTAATCGTTTTCACTGCCATTTCAGTCCGCGTGATTGGCAGCGTCTGATT
AATAACAATTGGGGTTTTCGTCCGAAACGCCTGAACTTTAAACTGTTTAATATCCAGGTGAAAGAAGTGACCCA
GAACGACGGCACCACCACCATTGCAAATAATCTGACCAGCACCGTTCAGGTTTTTACCGATAGCGAATATCAGC
TGCCTTATGTTCTGGGTAGCGCACATCAGGGTTGTCTGCCACCGTTTCCGGCAGATGTTTTTATGGTTCCGCAG
TATGGTTATCTGACCCTGAATAATGGTAGCCAGGCAGTTGGTCGTAGCAGCTTTTATTGTCTGGAATATTTTCC
GAGCCAGATGCTGCGTACCGGTAATAACTTTACCTTTAGCTATACCTTTGAGGATGTGCCGTTTCATAGCAGCT
ATGCACATAGCCAGAGCCTGGATCGTCTGATGAATCCGCTGATTGATCAGTATCTGTATTATCTGAGCCGTACC
AATACACCGAGCGGTACAACCACACAGAGCCGTCTGCAATTTAGTCAGGCAGGCGCAAGCGATATTCGTGATC
AGAGCCGTAATTGGCTGCCTGGTCCGTGTTATCGTCAGCAGCGTGTTAGCAAAACCAGCGCAGATAACAATAA
CAGCGAATATAGTTGGACCGGTGCCACCAAATATCATCTGAATGGTCGTGATAGCCTGGTTAATCCGGGTCCT
GCAATGGCCAGCCATAAAGATGATGAAGAAAAATTCTTTCCGCAGAGTGGCGTTCTGATTTTTGGTAAACAGG
GTAGCGAAAAAACCAACGTGGATATCGAAAAAGTGATGATCACCGATGAAGAAGAGATTCGTACCACCAATC
CGGTTGCGACCGAACAGTATGGTAGCGTTAGCACCAATCTGCAACGTGGTAAT**CATCATCACCATCATCAT**CG
TCAGGCAGCAACCGCAGATGTTAATACCCAGGGTGTTCTGCCTGGTATGGTTTGGCAGGATCGTGATGTTTAT
CTGCAAGGTCCGATTTGGGCAAAAATTCCGCATACCGATGGTCATTTTCATCCGAGTCCGCTGATGGGTGGTTT
TGGTCTGAAACATCCGCCTCCGCAGATTCTGATTAAGAATACTCCGGTTCCGGCAAATCCGAGCACCACCTTTA
GCGCAGCAAAATTTGCCAGCTTTATTACCCAGTATAGTACCGGTCAGGTTAGCGTTGAAATTGAATGGGAACT
GCAAAAAGAAAACAGCAAACGTTGGAATCCGGAAATTCAGTATACCAGCAACTATAACAAAAGCGTGAACGT
GGATTTTACCGTGGATACCAATGGTGTTTATAGCGAACCGCGTCCGATTGGCACCCGCTATCTGACACGTAATC
TGTAA

2.4 VP3 587His_6 amino acid sequence

MATGSGAPMADNNEGADGVGNSSGNWHCDSTWMGDRVITTSTRTWALPTYNNHLYKQISSQSGASNDNHYF
GYSTPWGYFDFNRFHCHFSPRDWQRLINNNWGFRPKRLNFKLFNIQVKEVTQNDGTTTIANNLTSTVQVFTDSEY
QLPYVLGSAHQGCLPPFPADVFMVPQYGYLTLNNGSQAVGRSSFYCLEYFPSQMLRTGNNFTFSYTFEDVPFHSSY
AHSQSLDRLMNPLIDQYLYYLSRTNTPSGTTTQSRLQFSQAGASDIRDQSRNWLPGPCYRQQRVSKTSADNNNSEY
SWTGATKYHLNGRDSLVNPGPAMASHKDDEEKFFPQSGVLIFGKQGSEKTNVDIEKVMITDEEEIRTTNPVATEQY
GSVSTNLQRGN**HHHHHH**RQAATADVNTQGVLPGMVWQDRDVYLQGPIWAKIPHTDGHFHPSPLMGGFGLKH

PPPQILIKNTPVPANPSTTFSAAKFASFITQYSTGQVSVEIEWELQKENSKRWNPEIQYTSNYNKSVNVDFTVDTNG
VYSEPRPIGTRYLTRNL

2.5 VP3 CTTEVHis$_6$ coding sequence

the C-terminal extension is in boldface

ATGGCAACCGGTTCAGGCGCGCCAATGGCAGATAATAATGAAGGTGCAGATGGTGTTGGTAATAGCAGCGGT
AATTGGCATTGTGATAGCACCTGGATGGGTGATCGTGTTATTACCACAAGCACCCGTACCTGGGCACTGCCGA
CCTATAATAACCATCTGTATAAACAAATTAGCAGCCAGAGCGGTGCAAGCAATGATAATCATTATTTTGGTTAT
AGCACCCCGTGGGGCTATTTTGATTTTAATCGTTTTCACTGCCATTTCAGTCCGCGTGATTGGCAGCGTCTGATT
AATAACAATTGGGGTTTTCGTCCGAAACGCCTGAACTTTAAACTGTTTAATATCCAGGTGAAAGAAGTGACCCA
GAACGACGGCACCACCACCATTGCAAATAATCTGACCAGCACCGTTCAGGTTTTTACCGATAGCGAATATCAGC
TGCCTTATGTTCTGGGTAGCGCACATCAGGGTTGTCTGCCACCGTTTCCGGCAGATGTTTTTATGGTTCCGCAG
TATGGTTATCTGACCCTGAATAATGGTAGCCAGGCAGTTGGTCGTAGCAGCTTTTATTGTCTGGAATATTTTCC
GAGCCAGATGCTGCGTACCGGTAATAACTTTACCTTTAGCTATACCTTTGAGGATGTGCCGTTTCATAGCAGCT
ATGCACATAGCCAGAGCCTGGATCGTCTGATGAATCCGCTGATTGATCAGTATCTGTATTATCTGAGCCGTACC
AATACACCGAGCGGTACAACCACACAGAGCCGTCTGCAATTTAGTCAGGCAGGCGCAAGCGATATTCGTGATC
AGAGCCGTAATTGGCTGCCTGGTCCGTGTTATCGTCAGCAGCGTGTTAGCAAAACCAGCGCAGATAACAATAA
CAGCGAATATAGTTGGACCGGTGCCACCAAATATCATCTGAATGGTCGTGATAGCCTGGTTAATCCGGGTCCT
GCAATGGCCAGCCATAAAGATGATGAAGAAAAATTCTTTCCGCAGAGTGGCGTTCTGATTTTTGGTAAACAGG
GTAGCGAAAAAACCAACGTGGATATCGAAAAAGTGATGATCACCGATGAAGAAGAGATTCGTACCACCAATC
CGGTTGCGACCGAACAGTATGGTAGCGTTAGCACCAATCTGCAACGTGGTAATCGTCAGGCAGCAACCGCAG
ATGTTAATACCCAGGGTGTTCTGCCTGGTATGGTTTGGCAGGATCGTGATGTTTATCTGCAAGGTCCGATTTGG
GCAAAAATTCCGCATACCGATGGTCATTTTCATCCGAGTCCGCTGATGGGTGGTTTTGGTCTGAAACATCCGCC
TCCGCAGATTCTGATTAAGAATACTCCGGTTCCGGCAAATCCGAGCACCACCTTTAGCGCAGCAAAATTTGCCA
GCTTTATTACCCAGTATAGTACCGGTCAGGTTAGCGTTGAAATTGAATGGGAACTGCAAAAAGAAAACAGCAA
ACGTTGGAATCCGGAAATTCAGTATACCAGCAACTATAACAAAAGCGTGAACGTGGATTTTACCGTGGATACC
AATGGTGTTTATAGCGAACCGCGTCCGATTGGCACCCGCTATCTGACACGTAATCTG**GGTCAGTTTTATCTGAA**
TGAACATCATCACCATCATCATTAA

2.6 VP3 CTTEVHis$_6$ amino acid sequence

MATGSGAPMADNNEGADGVGNSSGNWHCDSTWMGDRVITTSTRTWALPTYNNHLYKQISSQSGASNDNHYF
GYSTPWGYFDFNRFHCHFSPRDWQRLINNNWGFRPKRLNFKLFNIQVKEVTQNDGTTTIANNLTSTVQVFTDSEY
QLPYVLGSAHQGCLPPFPADVFMVPQYGYLTLNNGSQAVGRSSFYCLEYFPSQMLRTGNNFTFSYTFEDVPFHSSY
AHSQSLDRLMNPLIDQYLYYLSRTNTPSGTTTQSRLQFSQAGASDIRDQSRNWLPGPCYRQQRVSKTSADNNNSEY
SWTGATKYHLNGRDSLVNPGPAMASHKDDEEKFFPQSGVLIFGKQGSEKTNVDIEKVMITDEEEIRTTNPVATEQY
GSVSTNLQRGNRQAATADVNTQGVLPGMVWQDRDVYLQGPIWAKIPHTDGHFHPSPLMGGFGLKHPPPQILIK
NTPVPANPSTTFSAAKFASFITQYSTGQVSVEIEWELQKENSKRWNPEIQYTSNYNKSVNVDFTVDTNGVYSEPRPI
GTRYLTRNL**GQFYLNEHHHHHH**
